# Complex Protection of Some Steels in Sulfuric Acid Solutions by 1,2,4-Triazole Derivatives †

**DOI:** 10.3390/ma17194728

**Published:** 2024-09-26

**Authors:** Yaroslav G. Avdeev, Tatyana A. Nenasheva, Andrey Yu. Luchkin, Andrey I. Marshakov, Yurii I. Kuznetsov

**Affiliations:** A.N. Frumkin Institute of Physical Chemistry and Electrochemistry, Russian Academy of Sciences, 31 Leninskii Prospect, 119071 Moscow, Russiamar@ipc.rssi.ru (A.I.M.); yukuzn@gmail.com (Y.I.K.)

**Keywords:** corrosion, sulfuric acid, steel, corrosion inhibitors, hydrogen absorption by steel, kinetics of electrode reactions

## Abstract

The corrosion behavior of steels of various grades in sulfuric acid solutions with the addition of nitrogen-containing corrosion inhibitors has been studied. Compounds containing the 1,2,4-triazole moiety effectively protect low-carbon (St3, St20, 08PS), high-strength (70S2KhA), and stainless steels (1Kh18N9T) not only from corrosion but also from the hydrogen penetration into the metals in concentrated sulfuric acid solutions. In some cases, the degree of steel protection from corrosion by these compounds exceeded 99%. The possibility of creating mixed inhibitors for steel protection containing triazole derivatives and KI has been shown. The rate constants for the main steps of cathodic evolution and hydrogen penetration into steel in sulfuric acid solutions have been determined, and the subsurface concentrations of hydrogen in the metals have been calculated. Triazole derivatives were found to act as inhibitors of hydrogen absorption by steel in H_2_SO_4_ solution. The degree of protection of steel from hydrogen absorption can reach 97%. It has been shown that triazole derivatives act as complex inhibitors of steel corrosion in sulfuric acid solutions because, along with strong inhibition of metal corrosion, they prevent hydrogen absorption by steel.

## 1. Introduction

Sulfuric acid solutions are used to clean the surface of steel products from thermal scale, corrosion products, and various mineral deposits [1,2,3,4]. Upon contact of steels with sulfuric acid solutions, fast metal destruction occurs; therefore, these technological procedures require additional protection of metals. Acid cleaning of steels traditionally employs H_2_SO_4_ solutions with the addition of corrosion inhibitors. There is a broad range of corrosion inhibitors for protection of steels in acid solutions [5,6,7,8,9,10,11,12,13,14,15].

It has to be understood that during the corrosion of steels in acid solutions, metals can be destroyed not only due to the electrochemical reaction of the metal with the acid but also due to penetration of hydrogen into the metal bulk [16,17,18,19,20,21]. Absorption of hydrogen by the bulk of many steels can significantly change their mechanical properties. Thus, modern corrosion inhibitors should provide complex protection of steels, significantly slowing down the interaction of steels with the environment and preventing the absorption of released hydrogen. The hindrance of hydrogen absorption by steel due to corrosion inhibitors can be indirectly judged by the decrease in the rate of hydrogen penetration into a metal [22,23,24,25,26,27,28,29,30,31,32,33,34,35]. More complete information on the effect of corrosion inhibitors on the hydrogen absorption by steel can be obtained by direct determination of the hydrogen content in the metal by various methods, such as vacuum or electrochemical extraction. In parallel, it is possible to monitor the preservation of the metal’s mechanical properties [36,37,38].

Organic compounds of various natures [39,40,41,42] and green inhibitors, primarily plant extracts [43,44,45,46,47], are recommended for protecting steels in sulfuric acid environments (Table 1). These compounds are mainly proposed for protecting mild and carbon steels. Their use in dilute sulfuric acid solutions with a temperature close to room temperature is assumed. An increase in the temperature of the acid solution worsens the protective effect of these inhibitors. No information is provided on the suppression of hydrogen absorption by steel by these compounds. The data provided indicate the need to search for corrosion inhibitors capable of protecting steels of different chemical compositions in sulfuric acid solutions with different concentrations over a wide range of temperatures. In this case, the corrosion inhibitor should prevent the sorption of released hydrogen by steel.

The range of compounds for which the ability to provide complex inhibitor protection for various groups of steels in acid solutions has been studied is rather limited. Nitrogen-containing heterocycles, including triazole derivatives, are a promising group of inhibitors of steel corrosion in acid solutions [48,49,50]. In this work, it seemed important to study steel corrosion inhibitors capable of providing complex protection of steel in sulfuric acid solutions, such as 1,2,4-triazole derivatives with various structures.

## 2. Materials and Methods

### 2.1. Materials

The corrosion behavior of various iron alloys, such as low-carbon steels (St3, St20, and 08PS), high-strength steel (70S2KhA), and stainless steel (1Kh18N9T), is most often used as structural materials for equipment in contact with H_2_SO_4_ solutions. The chemical composition of these materials is presented in Table 2.

Three nitrogen-containing corrosion inhibitors were chosen as inhibitors of corrosion and hydrogen absorption by steels: compound I without a triazole ring ([C_n_H_2n+1_N^+^(CH_3_)_2_CH_2_C_6_H_5_]Cl, where n = 10–18) and two 1,2,4-triazole derivatives: [(C_6_H_5_CH_2_)_3_N^+^–C_2_H_2_N_3_]Cl^−^ (compound II) and [(C_8_H_17_)_3_N^+^–C_2_H_2_N_3_]Br^−^ (compound III).

Distilled water, concentrated sulfuric acid, potassium iodide, potassium bromide, and potassium rhodanide were used to make the solutions. All the reagents were of “chemically pure” grade. The solutions for electrochemical studies were deaerated with gaseous hydrogen or argon to remove dissolved molecular oxygen.

### 2.2. Methods

The measurements were carried out using instruments from the Center for Collective Use of Physical Research Methods in the A.N. Frumkin Institute of Physical Chemistry and Electrochemistry, Russian Academy of Sciences.

#### 2.2.1. Determination of Steel Corrosion Rate under Isobaric Conditions

The corrosion rate of steel was determined by the mass loss of metal samples as a result of their contact with an aggressive medium. To prevent evaporation of components of the aggressive environment, glass vessels for corrosion studies were equipped with a Graham condenser. Experiments were conducted in open vessels under isobaric conditions at atmospheric pressure. The corrosion rate was calculated using data on the mass loss of samples:

*ρ* = Δ *m*
*τ*
^−1^
*S*^−2^ (1)

where Δ *m* is the mass loss of a steel sample during corrosion studies; *τ* is the duration of the corrosion study; and *S* is the surface area of the steel sample.

For corrosion studies, coupons of St3, St20, 70S2KhA, 1Kh18N9T steels measuring 50 mm × 20 mm × 3 mm were used. For 08PC steel, coupons of 120 mm × 10 mm × 0.5 mm were used. The ratio of the solution volume to the surface area of the samples was 2.5 mL/cm^2^. At least 5 measurements were taken per experimental point. The experimental error did not exceed 7%. The general temperature range of solutions for corrosion studies was 25–100 °C.

The effect of individual substances and mixtures on steel corrosion was estimated by the inhibition coefficient, calculated as follows:

γ = *ρ*_o_
*ρ*_ad_^−1^ (2)

And the degree of protection, calculated as follows:

*Z* = [(*ρ*_o_
*− ρ*_ad_) *ρ*_ad_^−1^] 100% (3)

where *ρ*_ad_ and *ρ*_o_ are the corrosion rates in the medium with and without an additive, g/(m^2^·h).

#### 2.2.2. Determination of Steel Corrosion Rate from Autoclave Corrosion Test Data

**Figure 1:**
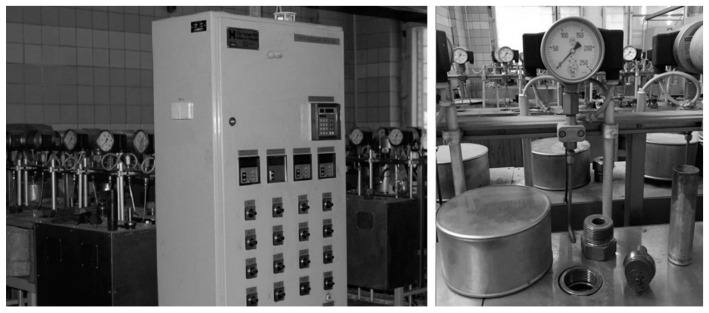
External appearance of the Huber autoclave.

The study of steel corrosion in sulfuric acid solutions under isochoric conditions was performed in Huber autoclaves with automatic control (Huber, Finland) (Figure 1). Quartz cells were used to isolate the acid solution from contact with metal parts of the autoclave. The corrosion rate of steel during autoclave corrosion tests was calculated using Equation (1). Equations (2) and (3) were used for quantitative characterization of the effect of individual substances and mixtures on steel corrosion.

#### 2.2.3. Vacuum Extraction Method

The content of absorbed hydrogen in metals was determined by measurement of hydrogen desorption under heating (500 °C) in a special vacuum unit. The hydrogen pressure in the unit was monitored by a McLeod mercury manometer. The hydrogen content in steel *V*(H_2_) (cm^3^/100 g of steel) was determined by the formula:

*V*(H_2_) = *F p*(H_2_) *m*^−1^ (4)

where *F* is the empirical constant characterizing the volume of the internal part of the unit; *p*(H_2_) is the pressure of desorbed gaseous hydrogen; and *m* is the mass of the steel specimen. The background hydrogen content was determined by subtracting the hydrogen from the samples not subjected to corrosion testing.

#### 2.2.4. Testing Steel Specimens for Cyclic Bending

The mechanical properties of steel were characterized by metal resistance to cyclic bending. Plasticity π was determined as the number of bends of the metal sample until fracture:

*π* = *β*_0_
*β*^−1^ 100% (5)

where *β* and *β*_0_ indicate the number of cyclic bends of steel specimens in contact and without contact with the corrosive medium.

#### 2.2.5. Potentiodynamic Polarization

Electrode reactions of steels in sulfuric acid solutions (60 °C) were studied using an EL 02.061 potentiostat (“ELNITEKS”, Moscow, Russia) with a dynamic potential scanning rate of 0.0005 V/s. The experiments were conducted in a thermostatically controlled glass electrochemical cell with separate spaces for the polarizing (smooth platinum) and reference electrodes (silver/silver chloride electrode). Coupons (5 mm × 5 mm × 0.5 mm) were used. Before recording the cathode and anodic polarization curves, the coupon was kept in the working solution until a constant value of the corrosion potential was established.

#### 2.2.6. Membrane Test

The effect of solution composition on the reaction rates of hydrogen evolution and penetration into the metal was studied in a Devanathan–Stachurski cell [51,52]. Steel membranes with a thickness of 0.1 mm and a working surface area of 4.25 cm^2^ were used as the working electrodes.

Both membrane sides were ground with SiC up to #600 grit, then chemically etched in 16% HCl for 1 min and thoroughly washed with distilled water. After that, a palladium film was electrochemically deposited on the membrane’s exit side for 100 s at a constant current density of 25 mA cm^−2^. The palladium plating solution contained 25 g L^−1^ PdCl_2_ and 20 g L^−1^ NH_4_Cl. The solution pH was adjusted to 8.5 by adding the required amount of NH_4_OH. Prior to the tests, the Pd-plated membranes were degassed at room temperature for at least 2 days.

The diffusion part of the cell was filled with 0.1 M NaOH, and the membrane was polarized at a potential of 0.45 V vs. SHE. The hydrogen flux at the exit surface was measured as its Faradic equivalent *i*_p_ = *i* − *i*_bg_, where *i*_bg_ is the background current density. The background current density was less than 5 × 10^−3^ A m^−2^. A constant potential (*E* = const) was applied to the working side of the membrane, and the external current (*i*_c_) was measured. The stationary current of hydrogen penetration through the membrane (*i*_p_) was recorded on the anodically polarized (diffusion) side of the membrane. The potential of the working membrane side was shifted from *E* = −0.4 V in the positive direction. To fill the hydrogen traps that exist both in the metal bulk and on its surface, before starting the measurements, a potential of −0.4 V was set on the working side of the membrane and maintained until a stationary value of the hydrogen penetration current was established (60 min). The cathodic curves and the *i*_p_-*E* plot were obtained at 20 mV increments and 30 min exposure time.

#### 2.2.7. IPZ Analysis

The cathodic hydrogen evolution reaction on iron and its alloys in acid media occurs by the discharge—chemical recombination mechanism, coupled rate control, or the slow discharge—irreversible chemical recombination mechanism [37,52,53]. IPZ analysis [53] was used to calculate the reaction constants of the main steps of cathodic discharge and hydrogen penetration into steel both in blank solutions and in solutions containing surface active compounds, e.g., corrosion inhibitors [52].

The IPZ analysis, which was used to calculate the rate constants of the steps of H^+^ ion discharge (*k*_1,i_), chemical recombination of hydrogen atoms (*k*_r_), kinetic diffusion constants (*k*), the degree of hydrogen coverage of the electrode surface (*θ*_H_), and the subsurface concentration of diffusion-mobile hydrogen in the metal phase ($C_{H}^{S}$) in solutions of organic corrosion inhibitors, is described in detail elsewhere [37].

#### 2.2.8. Determination of the Degree of Steel Protection against Hydrogen Absorption

The inhibitor efficiency was determined as the values of hydrogen content in the steel bulk (*V*(H_2_)) (method Section 2.2.4), calculated as follows:

(6) $$Z_{H}^{v}=[(V(H_{2})\;-\;{V(H_{2}),}_{inh})\;{V(H_{2})\;}^{-1}]100\%$$

and the molar concentration of hydrogen atoms under the steel surface ($C_{H}^{s}$) (method Section 2.2.7), calculated as follows:

(7) $$Z_{H}^{s}=\left[\left(C_{H}^{s}-C_{H,inh}^{s}\right)C_{H}^{s-1}\right]100\%$$

where *V*(H_2_) and *V*(H_2_),_inh_ indicate the hydrogen content in steel after exposure in the blank solution and in the solution containing an inhibitor, respectively; and $C_{H}^{s}$ and $C_{H,\;inh}^{s}$ are the subsurface concentrations of hydrogen in steel after exposure in the same respective solutions.

#### 2.2.9. Kelvin Probe Force Microscopy

Surface micrographs (400 × 600 μm) were taken using the built-in camera of a SolverNext II atomic force microscope (NovaPhotonix LLC, Saint Petersburg, Russia). Surface potential (VCPD) and surface topography were measured by double-pass Kelvin-probe force microscopy (KPFM) in amplitude modulation mode on the atomic force microscope under an open atmosphere. A silicon probe with a conductive platinum coating with a resonance frequency of 73 kHz and an elasticity coefficient of 4.5 N/m was used. The height of the second pass was 10 nm. The sample was grounded. Before the measurements, the probe was calibrated on a fresh surface of highly oriented pyrolytic graphite (HOPG).

The electron work function was calculated by the equation:

*V*pp = (*W*c − *W*s)/e (8)

where *V*pp is the measured surface potential; *W*c is the electron work function of the probe material; *W*s is the electron work function of the sample material; and *e* is the elementary electric charge.

## 3. Results and Discussion

### 3.1. Corrosion of Steels under Isobaric Conditions

Corrosion of St3 steel under isobaric conditions increases with an increase in temperature (Table 3). Under the conditions of the experiment, the maximum protection of steel in 2 M H_2_SO_4_ (25–80 °C) is provided by compound II and especially by compound III. Compound I inhibits St3 steel corrosion slightly less efficiently.

The corrosion of St3 steel in hot H_2_SO_4_ solutions increases with an increase in the acid content from 1 to 6 M (Table 4). Compounds III and II, which are derivatives of triazole, show high efficiency of such compounds for inhibiting metal corrosion. In contrast, in the presence of compound I, which does not contain a triazole moiety in its structure, the steel corrosion rates are higher than in the presence of compounds III and II.

For further research, a 2 M solution was chosen as the main concentration of sulfuric acid, since such solutions are most often used for pickling steel.

**Figure 2:**
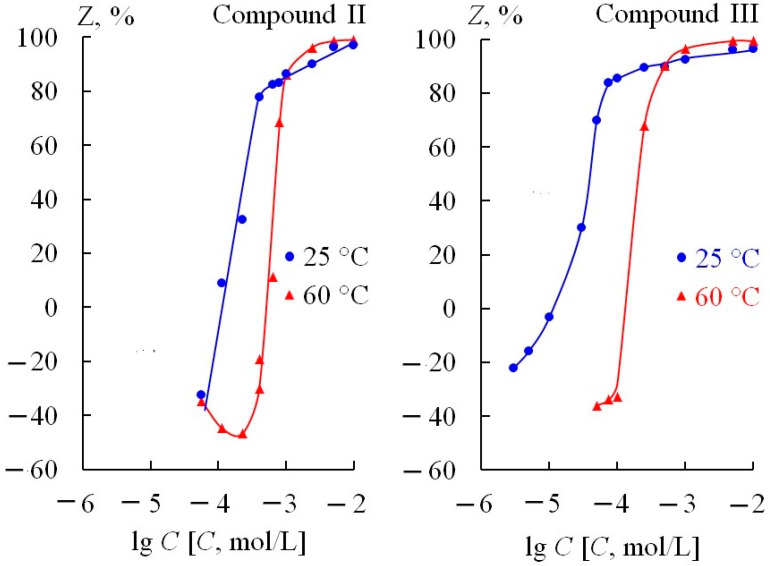
Degree of protection of St3 steel in 2 M H_2_SO_4_ vs. concentration of compounds II and III.

The 2 M concentration of sulfuric acid was chosen for more detailed studies. The effect of the content of compounds II and III on the corrosion rate of St3 steel in 2 M H_2_SO_4_ (60 °C) is presented in Figure 2. The plots of the degree of steel protection on the logarithm of concentration of compounds are S-shaped. At a low content of the compounds in the corrosive medium, they both stimulate corrosion. Compound I had shown to be the least effective in earlier tests and was therefore not tested in this study.

The possibility of increasing the protective effect of the organic compounds studied by combining them with salts of inorganic acids was studied. This approach is often used for the protection of metals in acid solutions by inhibitors [7,54,55,56]. It allows improving the protection of metal by inhibitors or reducing the consumption of an expensive component of the mixture by replacing it with a cheaper one. In this work, mixtures of organic compounds with KI, KBr, and KNCS were tested (Table 5). The highest protective effect is observed in mixtures of the organic compounds with KI (Z > 99% for all the mixtures). In all cases, the corrosion rate of St3 steel in the presence of mixed inhibitors is lower than in the presence of the individual components. The efficiency of all the mixed inhibitors decreases in the series: compound III > compound II > compound I. Of the mixtures containing KBr, the mixture with compound III (Z > 99%, too) is the most interesting. The lowest protective effects are observed for mixtures containing KNCS.

### 3.2. Corrosion of Steels under Isochoric Conditions

The technological processes for acid cleaning of steels can be carried out both in open systems (isobaric process) and in closed systems (isochoric process). The triazole derivatives studied in this work strongly inhibit the corrosion of steels under isobaric conditions. It was important to understand if they maintain this effect under isochoric corrosion conditions. These conditions can appear in technological production, for example, in the technological flushing of the internal surfaces of pipelines. To simulate this situation, corrosion tests were performed in autoclaves at high temperatures (80–100 °C). Under these conditions, the corrosion of St20 steel occurs at an extremely high rate (Table 6). At t = 100 °C, the rate can exceed 2 kg/(m^2^·h).

Under these experimental conditions, all the compounds under study inhibit the corrosion of St20 steel. All conditions being equal, the protective effect of the inhibitors decreases in the series: compound III > compound II > compound I. It should be noted that the protective effect of compound I at t = 100 °C is insufficient. In the presence of compound I, the corrosion rate of steel does not decrease below 26 kg/(m^2^·h), which is a poor result. The protective effect of compounds II and III is maintained even upon prolonged contact of steel with the acid up to 2 h, despite the harsh high-temperature corrosion conditions.

### 3.3. Effect of Organic Compounds on the Hydrogen Absorption by Steel

The organic compounds of the triazole series that we studied inhibit the corrosion of St3 and St20 steels in sulfuric acid solutions significantly under isobaric and isochoric conditions. It seems important to understand how these compounds inhibit the absorption of hydrogen by the corroding steel. Since high-strength steels are most prone to hydrogen embrittlement, 70S2KhA steel with a tensile strength of 880 MPa was chosen as the object of this study. Low-carbon steel 08PS and stainless steel 1Kh18N9T were studied for comparison.

The addition of compounds II and III protects the steel in 2 M H_2_SO_4_ (60 °C), reducing the corrosion rate 31- and 33-fold, respectively (Table 7). At the same time, the content of absorbed hydrogen in the metal is significantly smaller compared to the solution without additives. The degrees of hydrogen absorption inhibition are 96 and 95%, respectively. Under the same conditions, compound I falls behind compounds II and III in its ability to protect the metal and inhibit hydrogen absorption.

The triazole derivatives inhibit the corrosion of 08PS steel in 2 M H_2_SO_4_ (25 °C) more strongly than compound I (Table 8). Absorption of hydrogen by steel practically does not occur in the medium without the additives (Table 8). Compounds that inhibit the corrosion of steels often do not inhibit the absorption of hydrogen by the metal; moreover, they can even promote this process. It is crucially important that the organic additives studied in this work do not promote the absorption of hydrogen by the metal. During the corrosion of 08PS steel in a more heated sulfuric acid solution (60 °C), the absorption of hydrogen by the metal is significantly lower than by 70S2KhA steel. The absorption of hydrogen by the metal does not occur under these conditions in the presence of compounds II and III.

Technological equipment can be partially or entirely fabricated from stainless steels. Such alloys can be prone to corrosion in hot H_2_SO_4_ solutions (Table 9). Compounds II and III inhibit the corrosion of 1X18N9T stainless steel and hydrogen absorption by this steel. Compound I performs worse in the inhibition of these adverse processes.

Of the organic compounds under study, only those containing a triazole moiety are capable of providing complex protection of steels of various grades, preventing corrosion damage and hydrogen absorption by the metal. This is the important practical result that determines the need for a more detailed study of such compounds as inhibitors of acid corrosion of steels.

### 3.4. Effect of Organic Compounds on the Mechanical Properties of Steels

The accumulation of absorbed hydrogen by a metal results in significant loss of its mechanical properties. It is only compound III that significantly reduces the loss of ductility of 70S2KhA steel. This correlates well with the low content of absorbed hydrogen in the metal (Table 7). It should be noted that the ductility of the metal is completely lost in the presence of compound II, despite the low hydrogen content in the metal. This effect is largely due to the localization of the corrosion process on small areas of the metal surface by this additive, which adversely affects the mechanical properties of the metal.

### 3.5. Electrode Reactions of Steel

**Figure 3:**
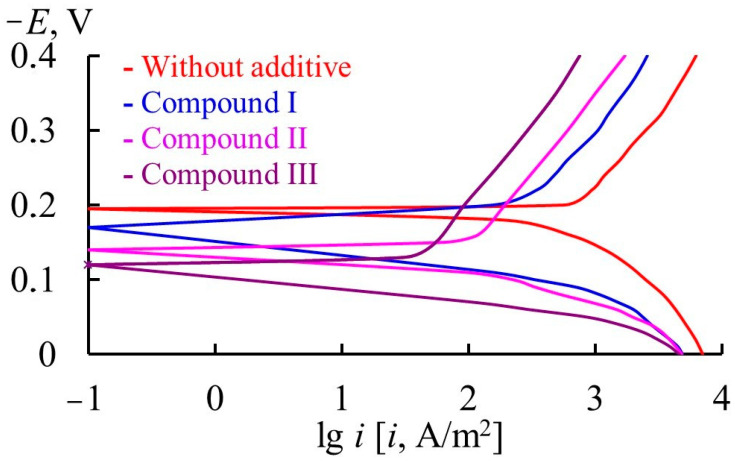
*E—*lg *i* plots of 70S2KhA steel in 2 M H_2_SO_4_ (60 °C).

To understand the nature of the protective effect of organic compounds on steel corrosion, their effect on the electrode reactions occurring on the metal should be studied. The corrosion of 70S2KhA steel in 2 M H_2_SO_4_ (60 °C) occurs in the potential region of active dissolution (Table 10, Figure 3). The slopes of cathodic and anodic polarization curves on steel are approximately 0.190 and 0.120 V, respectively, which is due to the processes of efficient sludge formation on the metal surface in the acid.

In the presence of all the organic compounds in question, the metal corrosion potential is shifted in the positive direction in comparison with that in the solution containing no additives. This indicates that the compounds preferentially inhibit the anodic reaction. In the presence of organic additives, the slopes of the cathodic polarization curves (*b*_c_) are slightly increased. It is somewhat unexpected that all the additives decrease the slope of the anodic polarization curves (*b*_a_). The observed effect is due to a significant reduction in sludge formation by these compounds at anodic polarization of the metal. The organic additives slow down the anodic reaction, and, as a result, the insoluble components of the alloy that form sludge are released on the surface more slowly. The inhibitory effect of the organic compounds on the cathodic and anodic reactions of steel decreases in the series: compound III > compound II > compound I, which correlates with the data on the corrosion of 70S2KhA steel obtained by the mass loss method.

**Figure 4:**
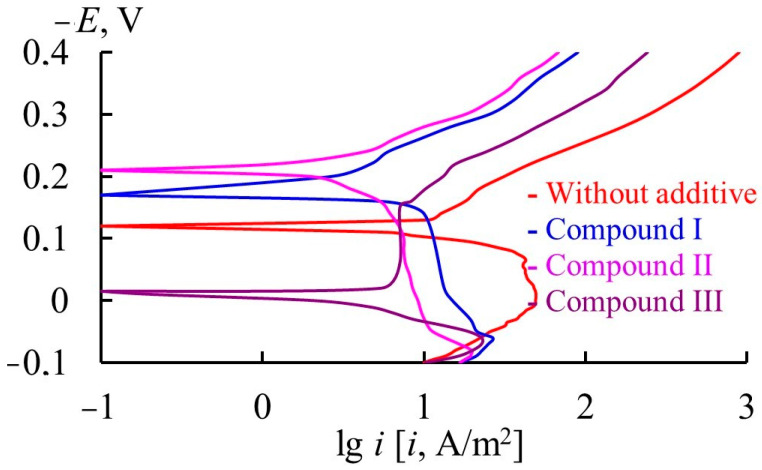
*E—*lg *i* plots of 11Kh18N9T steel in 2 M H_2_SO_4_ (60 °C).

In 2 M H_2_SO_4_ (60 °C), the corrosion potential of 1Kh18N9T stainless steel is in the region of active dissolution potentials (Table 10, Figure 4). Compounds II and I shift the corrosion potential to the negative region compared to the medium without the additives, which is typical of compounds that preferentially inhibit the cathodic reaction of the steel. The inhibiting effect of organic compounds on the anodic process of steel decreases in the series: compound III > compound II > compound I, while for the cathodic reaction, within the series: compound II > compound III > compound I. The overall result of this effect of triazole-containing compounds on the electrode reactions of stainless steel is a more significant inhibition of the general corrosion of the metal by compounds II and III in comparison with compound I.

The observed effect of significant inhibition of electrode reactions by compounds II and III is in many respects due to the presence of the triazole ring in their molecules, which provides the strongest binding of the inhibitor with the metal surface [37].

### 3.6. Kinetics of Reactions of Hydrogen Evolution and Penetration into the Metal

An average concentration value (5 mM) was chosen to study the effect of nitrogen-containing corrosion inhibitors on the kinetics of the cathodic process on steel in sulfuric acid. The studies were carried out in a Devanathan–Stachursky cell at room temperature (25 °C).

**Figure 5:**
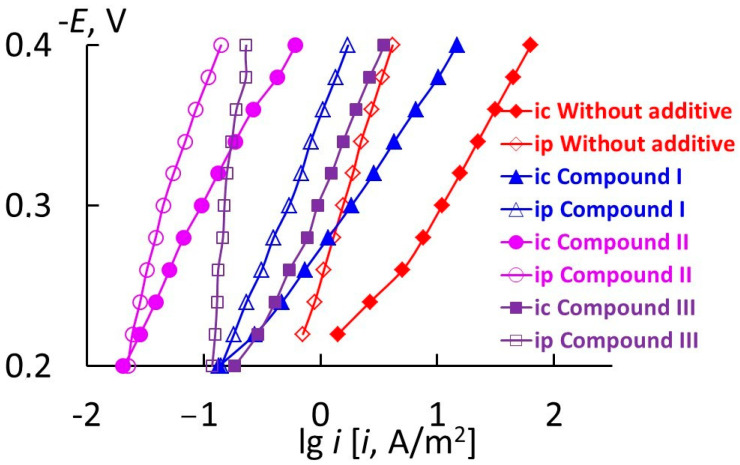
Cathodic polarization curves on St3 steel and plot of the current of hydrogen penetration vs. potential in solutions of 2 M H_2_SO_4_ containing corrosion inhibitors.

Figure 5 shows the cathodic polarization curves and plots of the rate of hydrogen penetration into steel vs. potential in sulfuric acid solution containing various additives. As it can be seen from the figure, the rates of hydrogen evolution (*i*_c_) and hydrogen penetration into the metal (*i*_p_) decrease in the presence of all three compounds in the entire region of potentials studied, but the degree of inhibition of the reactions is different. The effect is insignificant in the presence of compound I; the rate of the cathodic process decreases by a factor of six, and the rate of hydrogen penetration only by a factor of three. A more appreciable effect is observed for compound III (reduction of the cathodic current and hydrogen penetration current into the metal by an order of magnitude). According to the results of electrochemical studies, compound II is the most efficient inhibitor of both processes (*i*_c_ decreases 100-fold, while *i*_p_ decreases 35-fold).

To calculate the constants of the main steps of hydrogen evolution and penetration into the metal in media containing various additives, one has to know the degree of coverage of the metal surface with these additives (*θ*_inh_). The *θ*_inh_ values were determined from the decrease in the cathodic current. The calculated steady-state degrees of surface coverage were 0.83, 0.99, and 0.98 for compounds I, II, and III, respectively. These values were further used to calculate the constants of the main steps of hydrogen evolution and penetration into the metal.

### 3.7. Calculation of Kinetic Rate Constants of the Main Steps of Cathodic Evolution and Penetration of Hydrogen into Steel

IPZ analysis [37] allowed us to calculate the values of rate constants for hydrogen ion discharge (*k*_1,i_), chemical recombination of H atoms (*k*_r_), kinetic diffusion constants (*k*) from cathodic polarization curves, and the currents of hydrogen penetration into the metal (Figure 5). The main constants of the hydrogen evolution reaction in the presence of various additives and in the blank 2M H_2_SO_4_ solution were calculated (Table 11). As it can be seen in the table, all the compounds in question decrease the rate constants of H^+^ ion discharge and chemical recombination of H atoms and increase the kinetic diffusion constant.

Using the calculated constants (Table 11), the degree of coverage of the metal surface with hydrogen atoms (*θ*_H_), subsurface hydrogen concentration in the metal ($C_{H}^{s}$), and degree of protection of steel from hydrogen absorption ($Z_{H}^{s}$) were determined.

As one can see, addition of all the three compounds into sulfuric acid solution decreases both the amount of adsorbed (surface) hydrogen (*θ*_H_) and the concentration of hydrogen in the metal phase ($C_{H}^{s}$) (Table 11). All the compounds inhibit hydrogen adsorption in acidic media to varying degrees. The effect of compound I in 2M H_2_SO_4_ is insignificant: the amount of hydrogen in the metal bulk decreases only 3-fold. Upon addition of even insignificant amounts (5 mM) of compounds II and III to the acid solution, the degree of protection of steel from hydrogen absorption reaches almost 100%.

### 3.8. Atomic Force Microscopy (AFM)

The microphotographs, topographic maps of the surface, surface roughness values, and calculated work function values are presented in Table 12. The cantilever potential was 4.72 V.

According to the microphotograph of an 08PS steel sample, the surface was quite flat before treatment in the acid solution. This is confirmed by an analysis of the AFM image. There are some scratches on the surface no deeper than 7 nm, which were left after abrasive treatment. Analysis using the Gwyddion program allowed us to calculate the root mean square (RMS) roughness to be 3.37 nm. The average calculated electron work function is 4.323 eV.

Treatment of steel surface in 2 M H_2_SO_4_ without organic compounds results in the formation of a visually observable dense layer of etching sludge on the surface, which increases the initial roughness by an order of magnitude, up to 32 nm. Etched caverns up to 120 nm deep and clusters of sludge particles with up to 130 nm high can be distinguished on the AFM image. At the same time, the electron work function increases significantly in comparison to the blank solution. This is probably due to the dielectric properties of sludge on the sample surface.

Microphotographs of steel samples after treatment in 2 M H_2_SO_4_ containing organic additives show that there is significantly less sludge on the surface. This indirectly confirms the efficiency of metal protection by the additives studied. Analysis of AFM images shows that the average surface roughness increases nearly twofold compared to the initial sample. On the metal surface of samples treated with 2 M H_2_SO_4_ containing the additives, one can distinguish flows and drops that presumably consist of those additives. They are the smallest on the sample treated in 2 M H_2_SO_4_ with compound II and the largest in the case of compound III. This is indirectly confirmed by the energy diagrams showing that the surface potential on such areas is somewhat lower than on areas without the droplets. In the presence of all organic additives, the average work function increases as compared to the blank solution. However, it is impossible to conclude from these data which organic compound is more efficient for corrosion inhibition since the values are very close.

## 4. Conclusions

1. Organic compounds containing a triazole ring can efficiently protect low-carbon, high-strength, and stainless steels from corrosion and hydrogen absorption by the metals in sulfuric acid solutions (1–6 M) in a wide temperature range (25–100 °C) under isobaric and isochoric conditions. In some cases, the degree of protection of steels by these compounds exceeds 99%. The possibility of creating mixed inhibitors for steel protection containing a triazole derivative and a corrosion inhibitor has been shown.
2. Along with significant inhibition of steel corrosion in sulfuric acid solutions, triazole derivatives efficiently prevent hydrogen absorption by steel. In some cases, the degree of inhibition of hydrogen absorption reaches 100%. Compound III deserves special attention. While strongly inhibiting the corrosion of high-strength steel and hydrogen absorption, it preserves the mechanical properties of the metal.
3. It has been shown that the triazole derivatives inhibit both cathodic and anodic reactions on steels in sulfuric acid solutions at various temperatures.
4. The rate constants of the main steps of cathodic evolution of hydrogen and hydrogen penetration into steel have been determined, and the subsurface concentrations of hydrogen in the metal have been calculated. It has been found that all the compounds studied are inhibitors of hydrogen absorption by steel in H_2_SO_4_ solution. The highest effect was shown for compounds II and III that protect steel from hydrogen absorption ($Z_{H}^{v}$) by 97 and 94%, respectively.
5. Triazole derivatives are complex inhibitors of steel corrosion in sulfuric acid solutions since, along with strong inhibition of metal corrosion, they prevent the absorption of hydrogen by the metal. Triazole derivatives (compounds 2 and 3) show better results in comparison with organic and “green” corrosion inhibitors described in the scientific literature and recommended for protection of steels in sulfuric acid solutions. They do not lose their protective effect with temperature increase to 100 °C and can provide protection in a wide range of acid concentrations (1–6 M). Their protective effect is manifested for various grades of steel. It is important that these substances, when combined with potassium iodide, enhance their protective effect. This opens up opportunities to create mixed corrosion inhibitors with their participation, which significantly reduce the consumption of mixed components.

## Figures and Tables

**Table 1:** Protection of steels in H_2_SO_4_ solutions with various corrosion inhibitors.

No	Inhibitor	Corrosive Environment	Steel	*Z*, %	Ref.
1	10 mM 2-(1,5-Dimethyl-4-(2-methylbenzylidene)amino)-2-phenyl-1H-pyrazol-3(2H)-ylidene) hydrazinecarbothioamide	1 M H_2_SO_4_ (30–60 °C)	Mild steel	72.70–92.73	[39]
2	0.5 mM N-(4-(2-Aminothiazol-4-yl) phenyl) benzene sulfonamide	0.5 M H_2_SO_4_ (25–45 °C)	Mild steel	79.5–85.5	[40]
3	7.6 mM Indole	0.8 M H_2_SO_4_ (25–55 °C)	Carbon steel	41–81	[41]
4	1 mM (Z)−1-(5-nitro-2-oxoindolin-3-ylidene) thiosemicarbazide	0.5 M H_2_SO_4_ (25 °C)	Carbon steel	92.6	[42]
5	300 ppm organic-inorganic hybrid materials of 2,2′-dibenzimidazolyl butane dichlorhydrates and Na_4_P_2_O_7_	0.5 M H_2_SO_4_ (25 °C)	Mild steel	94.7	[43]
6	300 ppm Esomeprazole	1 M H_2_SO_4_ (30–60 °C)	Mild steel	70.66–80.97	[44]
7	500 ppm Cymbopogon Citratus extract	1 M H_2_SO_4_ (30–60 °C)	ASTM A572 steel	97	[45]
8	300 ppm Peanut shell extract	0.5 M H_2_SO_4_ (25 °C)	Carbon steel	95	[46]
9	0.1% Lilium brownii leaves extract	1 M H_2_SO_4_ (30–60 °C)	N80 steel	84.1–96.0	[47]

**Table 2:** Chemical composition of the steels under study. The content of chemical elements is given in mass %.

Chemical Elements	Steel Grade				
	St3	St20	08PS	70S2KhA	1Kh18N9T
C	0.14–0.22	0.17–0.24	0.05–0.11	0.65–0.75	≤0.12
Si	0.15–0.3	0.17–0.37	0.05–0.11	1.4–1.7	≤0.8
Mn	0.4–0.65	0.35–0.65	0.35–0.65	0.4–0.6	≤2.0
Ni	≤0.3	≤0.25	≤0.3	≤0.25	9–11
S	≤0.05	≤0.04	≤0.04	≤0.025	≤0.02
P	≤0.04	≤0.04	≤0.035	≤0.025	≤0.035
Cr	≤0.3	≤0.25	≤0.1	0.2–0.4	17–19
N	≤0.008	-	-	-	-
Cu	≤0.3	≤0.25	≤0.3	≤0.2	≤0.3
As	≤0.08	≤0.08	≤0.08	-	-
Ti	-	-	-	-	≤0.8
Fe	Balance				

**Table 3:** Parameters of St3 steel corrosion in 2 M H_2_SO_4_ at various temperatures.

Corrosion Parameters	Inhibitor (5 mM)			
	Without Inhibitor	Compound I	Compound II	Compound III
25 °C				
Corrosion rate, g m^−2^ h^−1^	12	2.8	0.42	0.44
Corrosion inhibition coefficient	-	4.3	29	27
60 °C				
Corrosion rate, g m^−2^ h^−1^	91	6.3	1.3	0.58
Corrosion inhibition coefficient	-	14	70	170
80 °C				
Corrosion rate, g m^−2^ h^−1^	420	9.5	3.2	1.8
Corrosion inhibition coefficient	-	44	130	230

**Table 4:** Parameters of St3 steel corrosion at various concentrations of H_2_SO_4_ at 60 °C.

Corrosion Parameters	Acid Concentration, M			
	1	2	4	6
Without inhibitor				
Corrosion rate, g m^−2^ h^−1^	70	91	210	290
Compound I				
Corrosion rate, g m^−2^ h^−1^	2.5	3.5	2.4	3.6
Corrosion inhibition coefficient	28	26	88	81
Compound II				
Corrosion rate, g m^−2^ h^−1^	2.2	1.3	1.5	1.8
Corrosion inhibition coefficient	32	70	140	160
Compound III				
Corrosion rate, g m^−2^ h^−1^	2.0	0.58	1.2	1.3
Corrosion inhibition coefficient	35	160	180	220

**Table 5:** Effect of salts on the parameters of St3 steel corrosion in 2 M H_2_SO_4_ (60 °C).

Corrosion Parameters	10 mM KAn *	Compound I		Compound II		Compound III	
		Mixture **	10 mM	Mixture **	10 mM	Mixture **	10 mM
Addition of KI							
Corrosion rate, g m^−2^ h^−1^	11	0.85	3.7	0.70	0.89	0.32	0.57
Corrosion inhibition coefficient	8.3	110	25	130	100	280	160
Addition of KBr							
Corrosion rate, g m^−2^ h^−1^	130	2.1	3.7	1.5	0.89	0.52	0.57
Corrosion inhibition coefficient	0.70	43	25	61	100	180	160
Addition of KNCS							
Corrosion rate, g m^−2^ h^−1^	54	2.6	3.7	1.1	0.89	0.92	0.57
Corrosion inhibition coefficient	1.7	35	25	83	100	99	160

* KAn = KI, KBr, and KNCS; ** 5 mM KAn + 5 mM Compound I, Compound II, and Compound III.

**Table 6:** Parameters of St20 steel corrosion in 2 M H_2_SO_4_ in tests under isochoric conditions.

Corrosion Parameters	Without Inhibitor	Compound I		Compound II		Compound III	
		10 mM	20 mM	10 mM	20 mM	10 mM	20 mM
Duration of experiments: 0.5 h. 80 °C							
Corrosion rate, g m^−2^ h^−1^	880	12	10	3.4	1.8	1.9	1.5
Corrosion inhibition coefficient	-	73	88	260	490	460	590
Duration of experiments: 1.0 h. 80 °C							
Corrosion rate, g m^−2^ h^−1^	840	11	9.8	2.0	1.3	1.3	1.1
Corrosion inhibition coefficient	-	76	86	420	650	650	760
Duration of experiments: 2.0 h. 80 °C							
Corrosion rate, g m^−2^ h^−1^	740	7.6	5.4	2.8	1.0	1.2	0.96
Corrosion inhibition coefficient	-	97	140	260	740	620	770
Duration of experiments: 0.5 h. 100 °C							
Corrosion rate, g m^−2^ h^−1^	2300	32	26	4.9	4.1	3.0	2.2
Corrosion inhibition coefficient	-	72	88	470	560	780	1000
Duration of experiments: 1.0 h. 100 °C							
Corrosion rate, g m^−2^ h^−1^	2000	27	25	4.1	3.5	2.6	1.9
Corrosion inhibition coefficient	-	74	80	490	570	770	1100
Duration of experiments: 2.0 h. 100 °C							
Corrosion rate, g m^−2^ h^−1^	1700	26	23	5.8	3.1	2.9	2.0
Corrosion inhibition coefficient	-	65	74	290	550	590	850

**Table 7:** Parameters of 70S2KhA steel corrosion in 2 M H_2_SO_4_. Duration of the experiments: 0.25 h (60 °C).

Corrosion Parameters	Inhibitor (5 mM)			
	Without Inhibitor	Compound I	Compound II	Compound III
Corrosion rate, g m^−2^ h^−1^	270	90	8.6	8.1
Corrosion inhibition coefficient	-	3.0	31	33
Volume of absorbed hydrogen in steel *, cm^3^/100 g of steel	3.41	1.58	0.15	0.17
Degree of inhibition of hydrogen absorption, %	-	54	96	95
Ductility of steel, %	**	-	5	93

* The data are corrected for metallurgical hydrogen that amounts to 0.34 cm^3^/100 g of steel. ** Complete loss of ductility of steel samples.

**Table 8:** Parameters of 08PS steel corrosion in 2 M H_2_SO_4_. Duration of the experiments: 2.0 h.

Corrosion Parameters	Inhibitor (5 mM)			
	Without Inhibitor	Compound I	Compound II	Compound III
Testing temperature—25 °C				
Corrosion rate, g m^−2^ h^−1^	12	2.0	0.22	0.23
Corrosion inhibition coefficient	-	6.0	55	52
Volume of hydrogen absorbed in steel *, cm^3^/100 g of steel	m.h. **	m.h.	m.h.	m.h.
Degree of inhibition of hydrogen absorption, %	-	-	-	-
Testing temperature—60 °C				
Corrosion rate, g m^−2^ h^−1^	250	14	0.65	0.68
Corrosion inhibition coefficient	-	18	380	370
Volume of hydrogen absorbed in steel, cm^3^/100 g of steel	0.67	0.36	m.h.	m.h.
Degree of inhibition of hydrogen absorption, %	-	46	~100	~100

* The data are corrected for metallurgical hydrogen that amounts to 0.57 cm^3^/100 g of steel. ** m.h.—the hydrogen content in steel samples does not exceed the metallurgical hydrogen value.

**Table 9:** Parameters of 1X18N9T steel corrosion in 2 M H_2_SO_4_. Duration of the experiments: 2.0 h (60 °C).

Parameters of Corrosion	Inhibitor (5 mM)			
	Without Inhibitor	Compound I	Compound II	Compound III
Corrosion rate, g m^−2^ h^−1^	50	2.0	0.40	1.2
Corrosion inhibition coefficient	-	25	130	42
Volume of hydrogen absorbed in steel *, cm^3^/100 g of steel	3.81	2.07	0.87	0.77
Degree of inhibition of hydrogen absorption, %	-	46	77	80

* The data are corrected for metallurgical hydrogen that amounts to 2.33 cm^3^/100 g of steel.

**Table 10:** Parameters of electrode reactions of 70S2KhA and 1X18N9T steels in 2 M H_2_SO_4_ (60 °C).

Inhibitor (5 mM)	*E*_cor_, V	*b*_c_, V	*b*_a_, V	*i*_c_ *, A/m^2^	*i*_a_ *, A/m^2^	*γ*_c_	*γ*_a_
70S2KhA steel							
Without inhibitor	−0.195	0.190	0.120	2500	2500	−	−
Compound I	−0.170	0.210	0.045	1050	335	2.4	7.5
Compound II	−0.140	0.220	0.040	606	225	4.1	11.1
Compound III	−0.120	0.220	0.030	293	15.6	8.5	160
1X18N9T steel							
Without inhibitor	−0.120	0.135	0.040	247	35.0	−	−
Compound I	−0.170	0.135	*i*_lim_ **	25.3	11.0	9.8	3.2
Compound II	−0.210	0.135	*i*_lim_	18,0	7.5	13.7	4.7
Compound III	−0.015	*i*_lim_	0.070	71.0	0.2 ***	3.5	175

* For 70S2KhA steel, the values *i*_c_ and *i*_a_ of cathodic and anodic current density are given at *E* = −0.30 and −0.10 V. For 1X18N9T steel, the values *i*_c_ and *i*_a_ are given at *E* = −0.30 and −0.08 V; ** *i*_lim_—limiting current; *** the value is found by extrapolating the first linear section (0.01–0.06 V) of the anodic polarization curve.

**Table 11:** Kinetic constants, degree of coverage of the metal surface with hydrogen atoms (*θ*_H_), subsurface concentration of diffusion-mobile hydrogen ($C_{H}^{s}$), and degree of steel protection from hydrogen absorption ($Z_{H}^{s}$) during cathodic polarization (*E* = −0.3 V) of St3 in 2 M H_2_SO_4_ containing various additives.

Additive	*k*_1,i_, mol m^−2^ s^−1^	*k*, m^3^ mol^−1^	*k*_r_, mol m^−2^ s^−1^	*θ*_H_ × 100	$C_{H}^{s}$, mol m^−3^	$Z_{H}^{v}$, %
2 M H_2_SO_4_	1.14 × 10^−4^	0.150	7.05 × 10^−2^	3.65	2.34 × 10^−1^	
+Compound I	1.91 × 10^−5^	0.375	2.11 × 10^−2^	2.19	7.20 × 10^−2^	69.2
+Compound II	9.94 × 10^−7^	3.690	9.46 × 10^−4^	1.61	6.39 × 10^−3^	97.3
+Compound III	9.74 × 10^−6^	1.390	1.35 × 10^−2^	1.80	1.47 × 10^−2^	93.7

**Table 12:** Surface parameters of 08PS steel based on the results of the Kelvin probe force microscopy method.

Surface Treatment	Microphotograph 400 × 600 μm	Topographic Map	Root Mean Square Roughness, s, nm	Work Function, Ws, eV
Without treatment	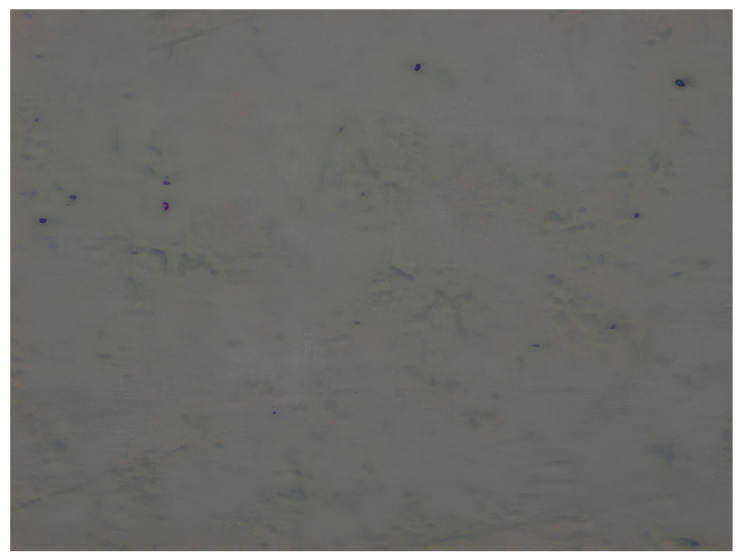	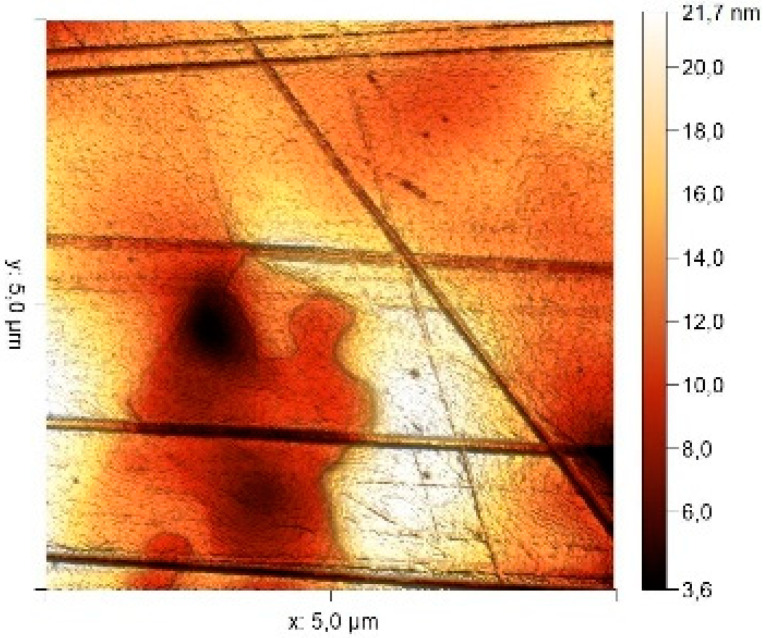	3.37	4.323
2 M H_2_SO_4_	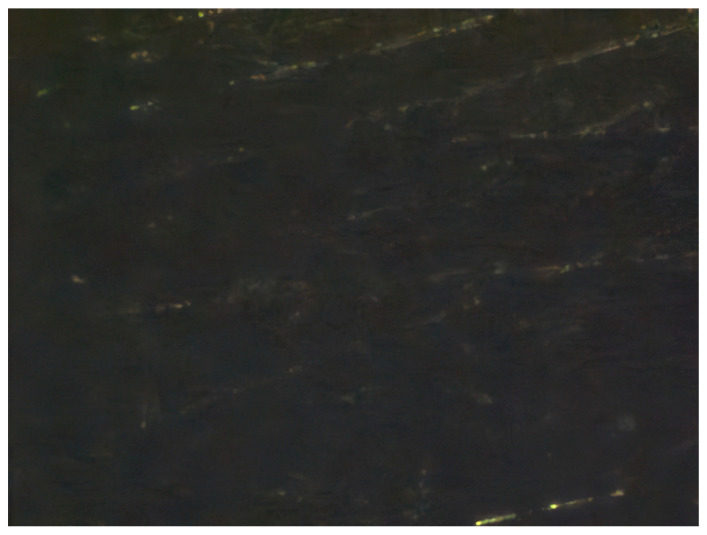	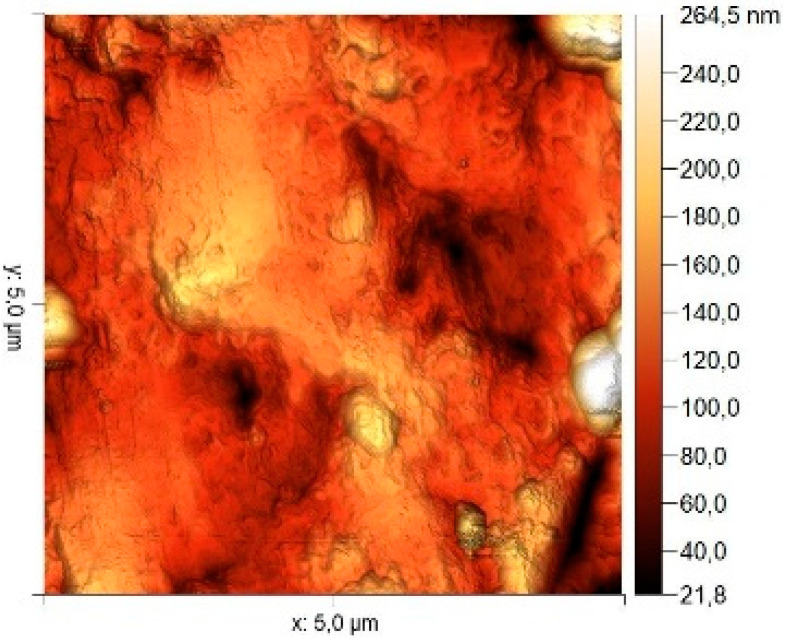	32	4.599
2 M H_2_SO_4_ + Compound I	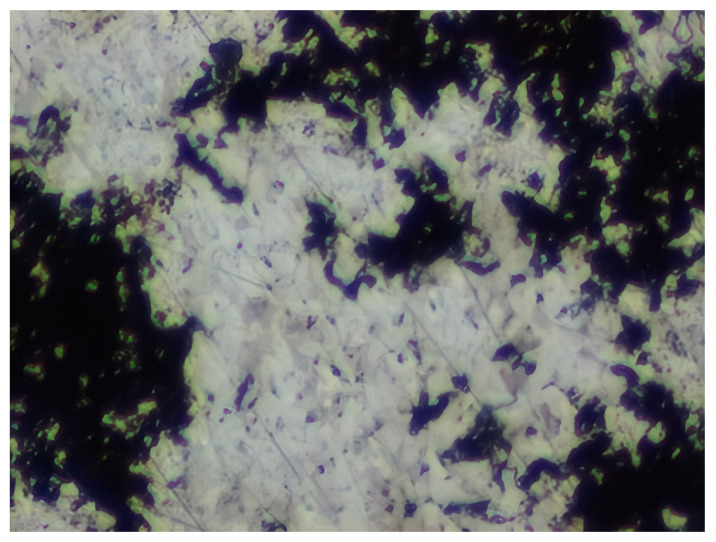	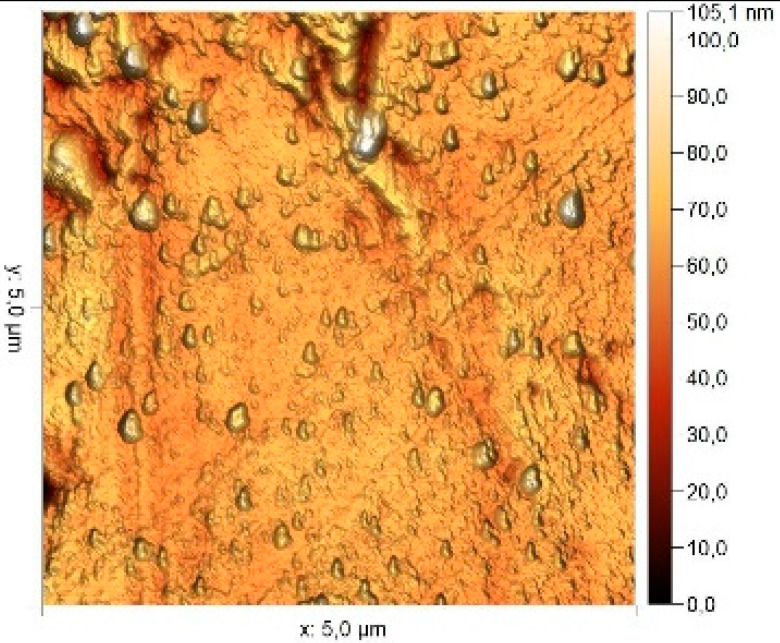	6	4.571
2 M H_2_SO_4_ + Compound II	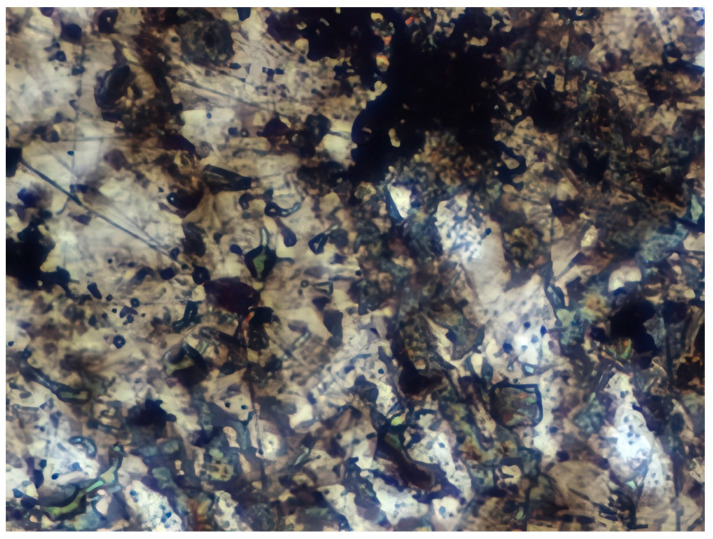	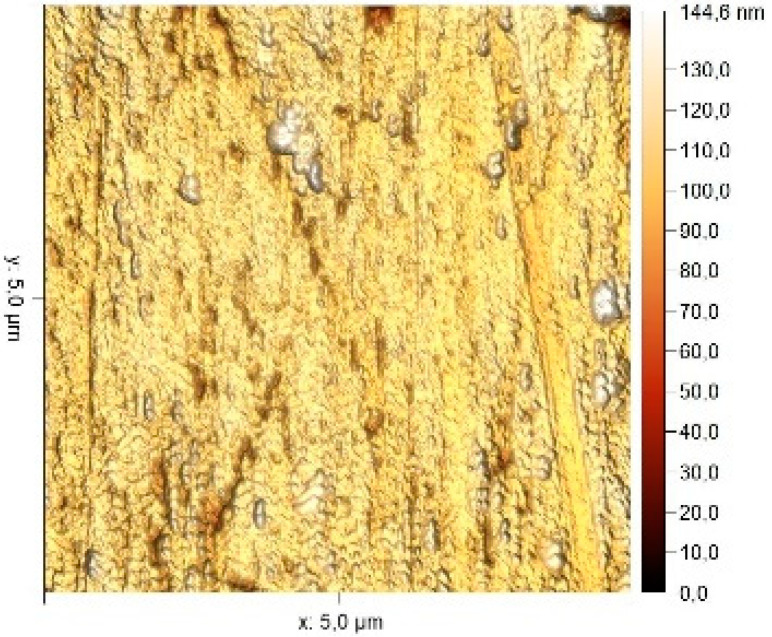	5.9	4.57
2 M H_2_SO_4_ + Compound III	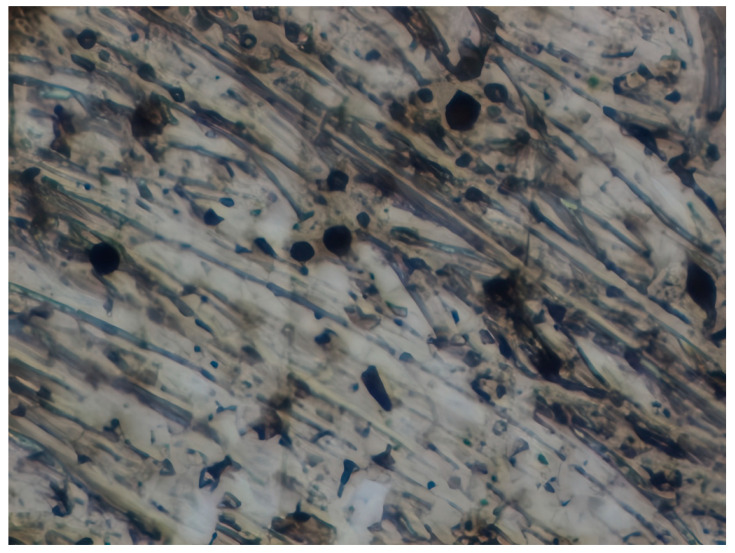	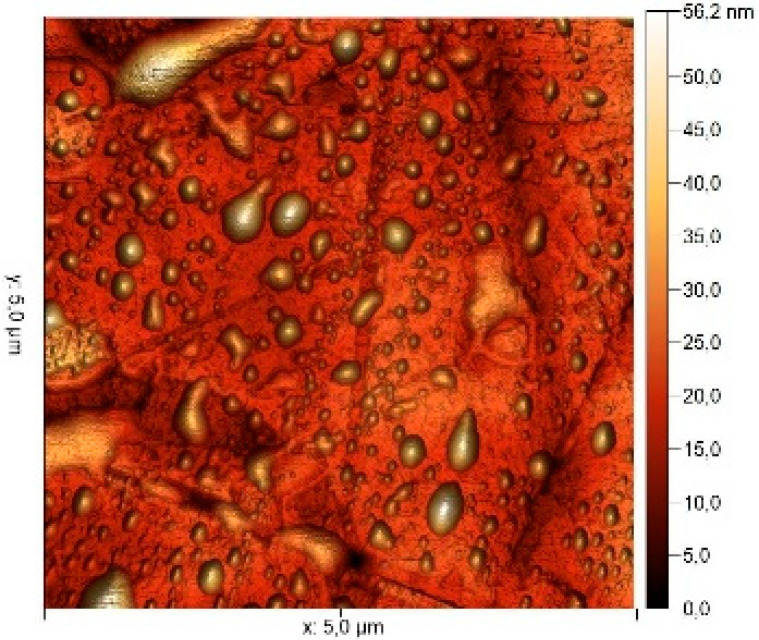	5.68	4.562

## Data Availability

The data are unavailable due to privacy or ethical restrictions.
